# Ultrasound guided external oblique intercostal plane block versus posterior transversus abdominis plane block for postoperative analgesia in adult patients undergoing open nephrectomy: a randomized trial

**DOI:** 10.1186/s12871-026-03935-4

**Published:** 2026-07-07

**Authors:** Amgad Elshikh, Lashin S. Ali, Mohammed S. ElSharkawy, Sameh M. Fathy, Bahaa G. Mohamed, Khaled Hamama, Mahmoud A. Elmohasseb, Eman H. Abu Shanab, Zainab T. Ragab, Saad A. Moharam, Ahmed F. Mady

**Affiliations:** 1 Anesthesiology, surgical ICU, and Pain management Department, Faculty of Medicine , Tanta University, Tanta, Egypt; 2 Basic Medical Science Department, Faculty of Dentistry, Al-Ahliyya Amman University, Amman, Jordan; 3 Anesthesia, ICU and Pain Management Department, South Egypt Cancer Institute, Assiut University, Assiut, Egypt; 4 Critical Care Department, King Saud Medical City, Riyadh First Health Cluster, Riyadh, Saudi Arabia

**Keywords:** External oblique intercostal plane block, Nephrectomy, Postoperative analgesia, Regional anesthesia, Transversus abdominis plane block, Ultrasound-guided, regional anesthesia

## Abstract

**Background:**

Nephrectomy procedures result in considerable postoperative discomfort, and limitations on the use of traditional management include opioid-related adverse effects and epidural-associated side effects. A prospective comparison was conducted to evaluate the relative effectiveness of two regional anesthetic techniques, the external oblique intercostal plane block (EOIPB) and the ultrasound-guided posterior transversus abdominis plane block (TAPB), in reducing postsurgical pain after open nephrectomy (OPN).

**Methods:**

Forty adults who underwent elective OPN were included in the study. The study population was randomly divided into two groups to receive either EOIPB or TAPB in a volume of 20 mL with 0.5% bupivacaine after the induction phase. The primary outcome was cumulative 24-hour pethidine consumption. Secondary outcomes included intraoperative fentanyl requirements, time to first rescue analgesia, pain scores at 0, 4, 8, 12, 18, and 24 h postoperatively, hemodynamic parameters, adverse events, and patient satisfaction.

**Results:**

The EOIPB group had significantly lower pain scores at 4, 8, 12, and 18 h after surgery compared to the TAPB group (*p* < 0.05). The EOIPB group used significantly less pethidine than the other group (61.5 ± 24.77 mg vs. 114 ± 12.31 mg, *p* < 0.001), and it took them longer to receive their first rescue analgesia (7.25 ± 0.91 vs. 6.1 ± 1.55 h, *p* = 0.007). The EOIPB group exhibited significantly higher patient satisfaction (*p* = 0.045), although the two groups had similar profiles of adverse events.

**Conclusions:**

In adults undergoing OPN, EOIPB provided superior early postoperative analgesia compared to TAPB during the first 18 h after surgery, as evidenced by lower pain scores, reduced opioid requirements, delayed time first to rescue analgesia, and higher patient satisfaction. Both blocks demonstrated comparable safety profiles.

**Clinical trial:**

Registration occurring at ClinicalTrials.gov (ID: NCT06452238), (URL: https://clinicaltrials.gov/study/NCT06452238?cond=NCT06452238&rank=1), Date: (30-5-2024).

## Background

Nephrectomy operations are also related to significant postoperative pain (POP), even with the development of minimally invasive laparoscopic procedures [1]. Adequate POP control is key for enhanced recovery, and more effective multimodal approaches are still warranted in clinical practice [2]. Pain management also merits special attention as a factor that significantly impairs the quality of life of a large segment of the adult population and remains one of the leading causes of disability, and personalized pain treatment regimens are an important goal [3].

The mainstays of modern multimodal pain treatment are currently patient-controlled opioid analgesia and epidural anesthesia, alone or in combination [4]. Opioid-based pain control is associated with a series of adverse effects, such as pruritus, PONV, and respiratory depression, and the latter two in particular are of significant concern due to reports of oversedation and apnea in at-risk populations like those with obstructive sleep apnea [5]. Epidural analgesia has also been linked to significant side effects like hypotension, postdural puncture headache, and restrictions in the use of anticoagulants, and rarer but serious complications like epidural hematoma, infection, and neurological injury are also safety concerns [6].

The TAPB is a regional anesthetic technique involving USG deposition of local anesthetic (LA) between the “internal oblique muscles (IOM)” and “transversus abdominis muscles (TAM)” [7–9], shown to be effective in the upper [10] and lower abdominal surgeries of the abdomen [11], and renal transplantation [12], with minimal complications [13]; employing subcostal, lateral, or posterior approaches [14]. The posterior technique offers superior analgesia for nephrectomy and renal transplantation [15].

The External Oblique Intercostal Plane Block (EOIPB), introduced by Elsharkawy et al. in 2021 [16], is a regional anesthetic technique in which local anesthetic is injected between the external oblique and intercostal muscles to target the thoracoabdominal nerves. It provides reliable analgesia of the upper lateral abdominal wall [17, 18], can be performed in the supine position unlike other blocks [19–21], and offers broader midline coverage compared to the serratus intercostal plane block [22].

This study aimed to compare the analgesic efficacy and safety of ultrasound-guided external oblique intercostal plane block and posterior transversus abdominis plane block in adult patients undergoing open nephrectomy.

Methods:

Forty adult subjects (≥ 18 years, ASA I or II, both sexes) scheduled for elective OPN at Tanta University Hospitals, Egypt, joined this prospective, randomized, controlled, double-masked trial from June 2024 to September 2025.

Approval for the investigation was granted by the institutional ethics committee, Faculty of Medicine, Tanta University, Tanta, Egypt (ID: 36264PR639/4/24), with registration occurring at ClinicalTrials.gov (ID: NCT06452238) (URL: https://clinicaltrials.gov/study/NCT06452238?cond=NCT06452238&rank=1), Date: (30-5-2024). This study was conducted in accordance with the Helsinki Declaration and adheres to CONSORT guidelines. All participants provided informed written consent.

Individuals were barred from involvement should they exhibit any of these states: established coagulation impairments, dermal abnormalities, or ongoing infection at the intended puncture location, known allergy to LA agents, pre-existing neurological disorders, history of substance abuse, body mass index exceeding 30 kg/m^2^, pregnancy, diabetic neuropathy, or severe cardiovascular pathology.

### Randomization and blinding

We used computer-generated randomization sequences (https://www.randomizer.org/) to hide the assignments, and each patient’s code was kept in a sealed, opaque envelope. The subjects were randomly allocated in a 1:1 ratio, using a parallel-group design, to two study arms (*n* = 20 each): Group EOIPB and Group TAPB. Due to the distinct anatomical landmarks and patient positioning, the anesthesiologist could not be blinded. All others were masked: patients were unaware of their allocation; postoperative assessors and research assistants were blinded to group; and the statistician analyzed de-identified data labeled as Group A and Group B. An independent anesthesiologist prepared 20 mL of 0.5% bupivacaine in an unlabeled syringe.

A comprehensive medical and surgical history was obtained from all participants. A physical examination was performed, along with standard laboratory tests.

A standard monitoring system was developed among all participants, including “pulse oximetry, electrocardiography, non-invasive blood pressure measurement, temperature monitoring, and capnography.” After the peripheral venous cannulation, a premedication dose of 2 mg intravenous midazolam was administered.

The induction of general anesthesia was performed according to a predetermined plan. Initially, 1.5–2.5 mg/kg propofol, respectively, and 1 µg/kg fentanyl were administered intravenously to initiate the induction procedure. After ascertaining the adequacy of anesthesia, 0.15 mg/kg cis-atracurium was administered intravenously to facilitate endotracheal intubation. A 2% solution of sevoflurane mixed with a 50% oxygen carrier gas was administered to maintain anesthesia. As required, 0.03 mg/kg cisatracurium was administered intravenously to relax the muscles further. The target end-tidal CO₂ pressure between 35 and 40 mmHg was maintained by finely regulating the mechanical ventilator parameters.

Guidelines for the safe maintenance of intraoperative hemodynamics were established as follows: In the presence of an increase in HR and MAP by more than 20% of the baseline value, 1 µg/kg of fentanyl was further administered via the IV line, after excluding all non-stimuli. The same surgical team performed all surgical procedures to minimize operator-related variability.

Hemodynamic parameters, including MAP and HR, were recorded at preoperative, immediately before block performance, and at 15-minute intervals throughout the surgical procedure until completion. Total intraoperative fentanyl consumption incidence was documented. Operative duration was recorded from skin incision to final wound closure.

All blocks were performed following GA induction, according to group allocation, using 20 mL of 0.5% bupivacaine.

### EOIPB technique

Elsharkawy et al.‘s [16] technique was applied. Subjects lay laterally with ipsilateral arm abduction, while the US probe was oriented cephalad-to-caudad paramedially along the anterior axillary line to locate the “external oblique muscle (EOM)” at T8. The probe was then advanced caudally to trace the EOM. At the subcostal level, it was rotated 90° to visualize the convergence of the IOM and TAM, then returned to the initial EOM point. The EOIP, located deep to the EOM and superficial to the sixth and seventh ribs, was identified. A needle was inserted in-plane, and the EOIP was hydrodissected with saline before LA administration.

### TAPB technique

Subjects were positioned supine. A high-frequency linear ultrasound probe (6–13 MHz) was placed transversely at the mid-axillary line, midway between the iliac crest and the costal margin. At the posterior axillary line between the 12th rib and iliac crest, the three lateral abdominal wall muscles (external oblique, internal oblique, and transversus abdominis) were identified, along with the anterior pole of the quadratus lumborum. A posterior approach to the TAPB was used. Using an in-plane technique, an 80 mm 22-gauge needle was advanced to the fascial plane between the internal oblique and transversus abdominis muscles, adjacent to the quadratus lumborum. After negative aspiration, 1 mL of saline was injected to confirm hydrolocalization, followed by a slow injection of 20 mL of 0.5% bupivacaine, with ultrasound confirmation of hypoechoic spread within the transversus abdominis plane.

Upon completion of the operative intervention, IV neostigmine (0.08 mg/kg) and atropine (0.02 mg/kg) were administered to counteract residual neuromuscular inhibition. The anesthetic drugs were then stopped. Following fulfillment of the extubation criteria and return to full consciousness, subjects were transferred to the post-anesthesia care unit (PACU).

### Postoperative management

A standardized multimodal analgesic regimen was administered. Paracetamol 1 gram intravenously every six hours as primary analgesia was given to all patients. As rescue analgesia, the active ingredient was 30 mg of pethidine, administered if the Numeric Rating Scale score was greater than 3, with repeat doses allowed if pain persisted after 30 min, until the Numeric Rating Scale score was less than 4.

Pain intensity was measured using the “Numerical Rating Scale” at fixed times: immediately upon arrival in the PACU (0 h) and at 4, 8, 12, 18, and 24 h postoperatively. The period between the end of surgery and the first rescue analgesic dose was carefully recorded, starting at the end of surgery and including the initial dose of pethidine administered as rescue analgesia. The total pethidine consumption for each patient over 24 h was recorded. Accordingly, All pethidine doses were converted to oral morphine milligram equivalents (MME) using an accepted equianalgesic conversion ratio (1 mg pethidine ≈ 0.3 mg oral morphine).

Patient satisfaction with the effectiveness of POP protocols was measured 24 h following their execution. This satisfaction was measured using a 5-point Likert scale, which has been shown to have psychometric properties [23]. The scale was carefully categorized so that 1 indicated extreme dissatisfaction, 2 indicated dissatisfaction, 3 indicated neutrality, 4 indicated satisfaction, and 5 indicated extreme satisfaction.

Adverse events were prospectively identified throughout the perioperative period. Hypotension, defined as a > 20% decrease in MAP from baseline, was treated with intravascular fluid boluses. Bradycardia, a > 20% decrease in HR from the reference value, was treated with an intravenous infusion of atropine 0.02 mg/kg body weight. Respiratory depression, indicated by oxygen saturation values of less than 95% with a requirement for oxygen therapy, was identified in this study. The incidence and grade of PONV were identified as adverse events and managed in accordance with institutional procedures. Other adverse events, including all complications possibly related to regional anesthesia and surgery, were identified as per this study.

The primary outcome measure was cumulative pethidine consumption over 24 h. Secondary outcomes included intraoperative incidence of fentanyl, time to rescue analgesia, pain scores, and intraoperative hemodynamic parameters.

### Sample size calculation

The sample size calculation was done by G*Power 3.1.9.2 (Universität Kiel, Germany). We performed a pilot study (5 cases per group) and found that the mean (± SD) cumulative consumption of pethidine over 24 h was 84 ± 13.4 in the EOIPB group and 102 ± 16.4 in the TAPB group. The sample size was based on the following considerations: 1.2 effect size, 95% confidence level, 90% power, a 1:1 group ratio, and adding 4 cases to each group to account for dropout. Therefore, we recruited 20 patients in each group.

### Statistical analysis

SPSS version 29 (IBM©, Armonk, NY, USA) was employed for data analysis. The Shapiro–Wilk test and histogram inspection were used to assess the normality of the data. Unpaired t-test analyzed parametric data (mean ± SD), Mann–Whitney test evaluated non-parametric data (median, IQR), and Chi-square or Fisher’s exact test compared categorical variables (frequency, %). *P* ≤ 0.05 indicated significance.

## Results

**Fig. 1:**
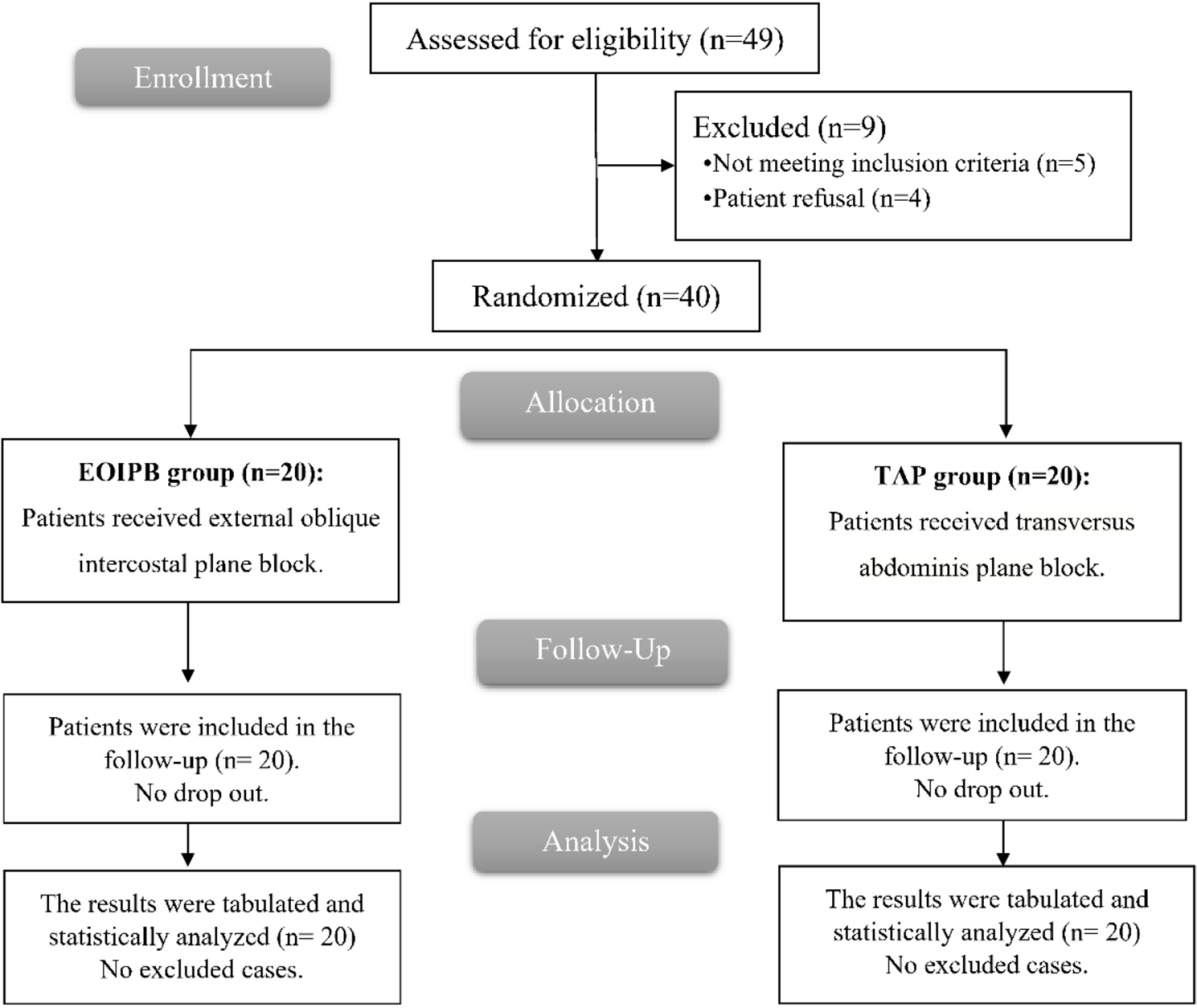
CONSORT flowchart of the enrolled patients

Of the 49 subjects assessed, five were ineligible, and four refused to participate. Forty subjects were randomized equally into two groups (20 subjects per group), followed up, and analyzed. Figure 1.

Demographics and surgery duration were similar across groups. Table 1.

**Table 1:** Demographic data and duration of surgery

		EOIPB group (*n* = 20)	TAPB group (*n* = 20)	*P* value	MD/RR (95%CI)
Age (years)		47.15 ± 10.72	44.1 ± 11.07	0.382	3.05(-3.93: 10.03)
Sex	Male	13 (65%)	11 (55%)	0.519	1.18(0.71:1.97)
	Female	7 (35%)	9 (45%)		
Weight (kg)		66.45 ± 9.8	69.65 ± 8.98	0.288	-3.2(-9.22 : 2.82)
Height (cm)		165.8 ± 7.67	167.05 ± 6.6	0.584	-1.25(-5.83 : 3.33)
Body mass index (kg/m^2^)		24.16 ± 2.93	25.06 ± 3.74	0.400	-0.91(-3.06 : 1.25)
ASA physical status	I	7 (35%)	9 (45%)	0.519	0.78(0.36:1.68)
	II	13 (65%)	11 (55%)		
Duration of surgery (min)		97.25 ± 8.66	101.25 ± 13.85	0.280	-4(-11.39 : 3.39)

Data expressed as mean ± SD or frequency (%)

*MD* Mean differences, *RR* Relative risk, *CI* Confidence interval

**Fig. 2:**
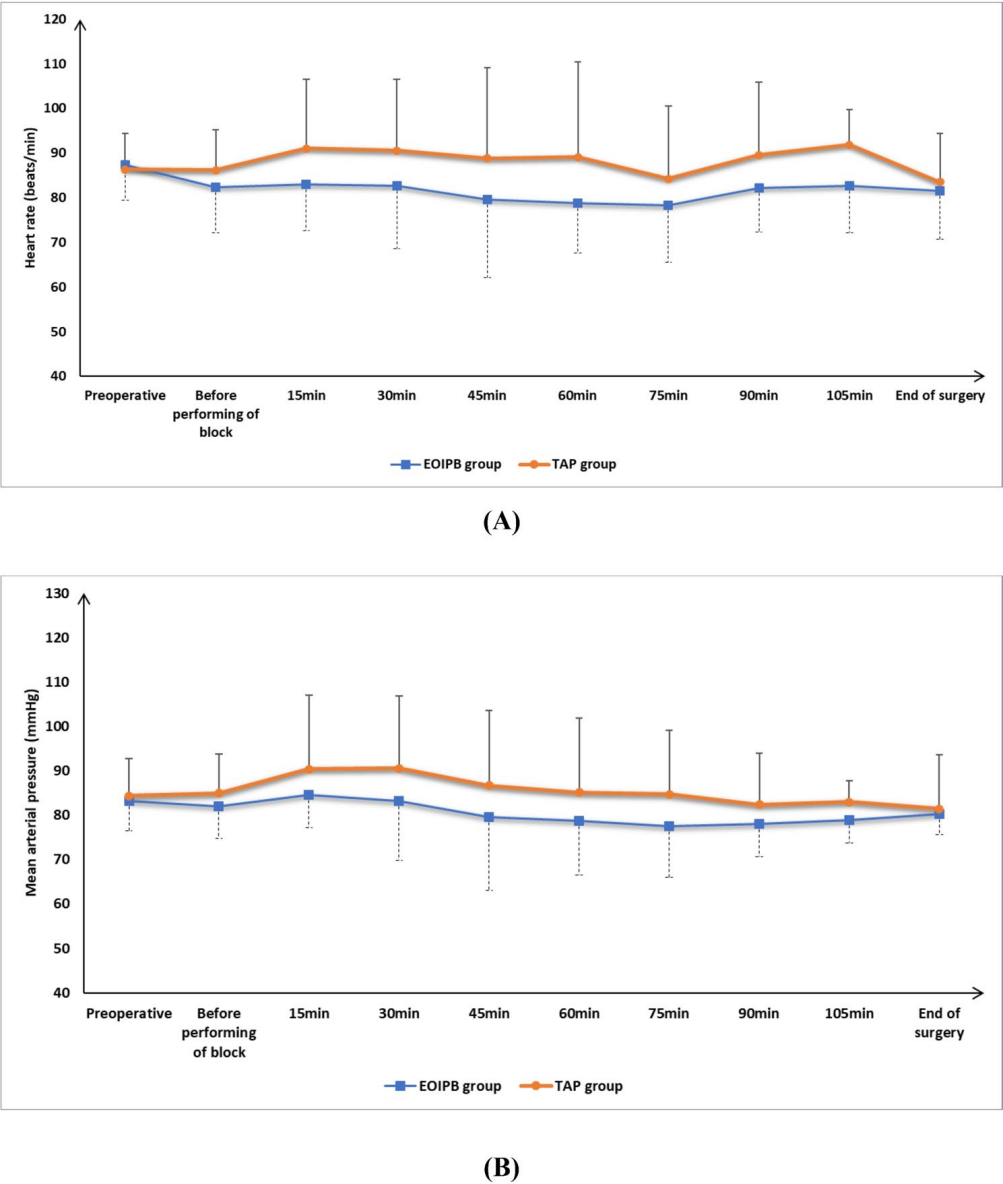
(**A**) Heart rate and (**B**) mean arterial blood pressure changes

Both groups’ HR and MAP measurements were similar at preoperative, before block, 15, 30, 45, 60, 75, 90, and 105-minute time points, as well as after the operation. Figure 2.

The NRS score was insignificantly different between the two groups at PACU and 24 h postoperatively. However, it was notably lower at 4, 8, 12, and 18 h in the EOIPB group compared to the TAPB group (*P* < 0.05). Table 2.

**Table 2:** Numerical rating scale (NRS) score

	EOIPB group (*n* = 20)	TAPB group (*n* = 20)	*P* value	MD(95%CI)
PACU	1(0–2)	1.5(0.75–2)	0.195	0(0–1)
4 h	1(1–2)	2(1.75–3.25)	< 0.001	2(1–3)
8 h	1(1–4)	3(2.75–4.5)	0.044	2(0–2)
12 h	3(2–4.25)	6(3–7)	0.018	2(0–3)
18 h	3(2–5)	5(3–6)	0.035	2(0–3)
24 h	3(2–6)	3.5(2–5)	0.989	0(-1-1)

Data expressed as median (IQR)

*NRS* Numerical rating scale, *MD* Median differences, *CI* Confidence interval

In both groups, intraoperative fentanyl use was similar. The EOIPB group requested rescue analgesia later than the TAPB group (*P* = 0.007). The EOIPB group consumed less opioid in the first 24 h compared to the TAPB group (*P* < 0.001). Table 3.

**Table 3:** Intraoperative and postoperative opioid use

	EOIPB group ** (** ***n*** ** = 20)**	TAPB group ** (** ***n*** ** = 20)**	*P* value	MD/RR (95%CI)
Intraoperative fentanyl consumption	1 (5%)	4 (20%)	0.741	0.368(0.06:2.18)
Time to first request of rescue analgesia (h)	7.25 ± 0.91	6.1 ± 1.55	0.007	1.15(0.34 : 1.96)
Total pethidine consumption in the first 24 h (mg)	61.5 ± 24.77	114 ± 12.31	< 0.001	-52.5(-65.02 : -39.98)
Morphine equivalents in the first 24 h (mg)	18.45 ± 7.43	34.2 ± 3.69	< 0.001	-15.75 -19.51 to -11.99)

Data expressed as mean ± SD or frequency (%)

MD Mean differences, RR Relative risk, CI Confidence interval

Both groups had similar bradycardia, hypotension, and PONV rates. No patient in either group had respiratory depression. The EOIPB group demonstrated greater patient satisfaction than the TAPB group (*P* = 0.045). Table 4.

**Table 4:** Side effects and patient satisfaction

		EOIPB group (*n* = 20)	TAPB group (*n* = 20)	P value	RR (95%CI)
Side effects	Bradycardia	2 (10%)	4 (20%)	0.774	0.5(0.1:2.43)
	Hypotension	4 (20%)	5 (25%)	0.362	0.8(0.25:2.55)
	PONV	2 (10%)	4 (20%)	0.774	0.5(0.1:2.43)
	Respiratory depression	0 (0%)	0 (0%)	---	**---**
Patient satisfaction	Extremely satisfied	13 (65%)	4 (20%)	0.045	**---**
	Satisfied	5 (25%)	8 (40%)		
	Neutral	2 (10%)	5 (25%)		
	Unsatisfied	0 (0%)	2 (10%)		
	Extremely dissatisfied	0 (0%)	1 (5%)		

Data expressed as frequency (%)

RR Relative risk, CI Confidence interval

## Discussion

The optimal management of POP is an important determinant that continues to influence surgical outcomes, with inadequate analgesic pain being a crucial factor associated with the development of chronic pain among patients [24]. USG fascial plane blocks have now been incorporated as a strategy associated with multimodal analgesic regimens, which are now identified as important approaches aimed at reducing opioid analgesic consumption while improving surgical outcomes [25].

The results indicated that subjects administered EOIPB had a significantly lower POP score than those administered TAPB, suggesting a meaningful analgesic advantage related to OPN.

The results are confirmed by a study by Shrey et al. [26], which found significantly greater pain relief at 30 min and 6 h postoperatively in EOIPB patients undergoing subcostal incisions compared with those undergoing subcostal TAPB, thereby further generalizing EOIPB’s advantage over upper abdominal analgesic procedures.

Although NRS differences were statistically significant at 4, 8, 12, and 18 h (1–3 points), clinical relevance depends on the minimal clinically important difference (MCID), estimated at 1.5–2 points on an 11-point scale [27]. Observed differences (1, 2, 3, and 2 points) met or exceeded this threshold at most time points, indicating clinically meaningful benefit. This clinical relevance is further supported by the significantly reduced opioid consumption and higher patient satisfaction in the EOIPB group.

EOIPB was found to offer better blockade between T4 and T10 by Rajitha et al. [25]. Conversely, TAPB predominantly affects the lower thoracic and upper lumbar dermatomes, an advantageous position that makes it ideal for nephrectomy, a procedure requiring analgesia between T6 and T10 [28], and this was further ratified by the shallow position of EOIP, making it ideal for blocking the anterior and lateral thoraco-abdominal nerve roots [29], mechanistically validated by Fujino et al. [30], whose study confirmed the sensory blockade between T8 and T12 nerve roots in cases involving robot-assisted partial nephrectomy procedures.

The significantly lower total consumption of pethidine medication among patients in the EOIPB group represents the meaningful opioid-sparing effect achieved, leading to better recovery and fewer opioid side effects. This concordance with Kavakli et al. [31] reported notably lower median opioid consumption at 24 h in bilateral EOIPB subjects undergoing laparoscopic sleeve gastrectomy (7.5 mg versus 14 mg, *p* = 0.0001), while Amin et al. [32] documented reduced opioid consumption in open supraumbilical procedures for EOIPB than TAPB. While Hong et al. [33] indicated that TAPB meaningfully lowered 24-hour opioid use versus systemic analgesia in laparoscopic nephrectomy.

The delay to the first rescue analgesia dose in EOIPB versus TAPB reflects prolonged effectiveness into the intermediate period, as proposed by Shrey et al. [26], which demonstrated a significantly greater mean delay to patient-controlled analgesia trigger (*p* = 0.001). The greater delay to the first rescue analgesia dose with EOIPB compared to TAPB was confirmed by Chopra et al. [34] as a component of bilateral right-sided EOIPB + posterior TAPB in laparoscopic cholecystectomy procedures (12.80 vs. 5.96 h, respectively; *p* < 0.001). The longer duration thereof, we speculate, is due to a depot effect within the fascial plane, augmented by neural exposure as proposed by Kamel et al. [35] and Tang et al. [36], which yields opioid-sparing action and a lower dose requirement for rescue analgesia in major abdominal surgery procedures.

Both approaches yielded similar values for hemodynamic parameters and safety profiles regarding the incidence of bradycardia, hypotension, and PONV, and the absence of respiratory depression, as reported in the literature on truncal fascial plane blocks, with low complication rates in each study. The surface anatomic plane between EOM and external intercostal muscles, presented by EOIPB, offers theoretical safety benefits due to a lower risk to adjacent structures, including vessels and the pleura [24, 31, 34, 36].

The EOIPB group’s significantly greater patient satisfaction ratings are a reflection of the cumulative effect related to decreased opioid use and better pain control, as seen in Shrey et al. [26]. The results provide sufficient evidence to consider including EOIPB as a component of multimodal analgesia regimens in OPN, as practiced in surgical recovery enhancement approaches. The highest preference is given to regional analgesic approaches, which reduce opioid use [25].

The results of this single-center study may be limited in generalizability, as it involved a small number of participants and stringent exclusion criteria. The natural variability in block procedures and the body areas targeted may make full blinding of the anesthesiologists a challenge, potentially leading to performance bias. The blocks were performed after general anesthesia, preventing sensory testing to confirm dermatomal spread and real-time block success. Thus, differences in analgesia may reflect technical variability rather than actual block effects. To reduce bias, experienced anesthesiologists used standardized ultrasound guidance, consistent drug volumes and concentrations, and consistent equipment. Moreover, the study may lack sufficient statistical power to detect meaningful effects for the secondary outcomes.

## Conclusions

EOIPB provides superior early postoperative analgesia compared with TAPB after OPN. Patients experienced significantly lower pain scores during the first 18 h, longer time first to rescue analgesia, and reduced 24-hour opioid consumption. Hemodynamic stability and adverse events were comparable between groups. Higher patient satisfaction with EOIPB likely reflects improved early pain control and decreased opioid exposure. EOIPB appears to be a valuable component of multimodal and enhanced recovery protocols, although benefits were limited to the early postoperative period, and long-term outcomes were not evaluated. Further research with larger sample sizes and extended follow-up is warranted to confirm these findings and evaluate sustained outcomes.

## Data Availability

Data is available on reasonable requests from the corresponding author.
